# Relationships between physical qualities and key performance indicators during match-play in senior international rugby union players

**DOI:** 10.1371/journal.pone.0202811

**Published:** 2018-09-12

**Authors:** Daniel J. Cunningham, David A. Shearer, Scott Drawer, Ben Pollard, Christian J. Cook, Mark Bennett, Mark Russell, Liam P. Kilduff

**Affiliations:** 1 Applied Sport Technology Exercise and Medicine Research Centre (A-STEM), College of Engineering, Swansea University, Swansea, Wales; 2 School of Psychology and Therapeutic Studies, University of South Wales, Rhondda Cynon Taff, Wales; 3 Welsh Institute of Performance Science, College of Engineering, Swansea University, Swansea, Wales; 4 Sky Performance Hub, Team Sky, Bath, England; 5 Saracens RFC, North London, London, England; 6 The Rugby Football Union, Greater London, London, England; 7 School of Social and Health Sciences, Leeds Trinity University, Leeds, England; Berner Fachhochschule, SWITZERLAND

## Abstract

The use of physical tests to profile physical capabilities, and provide training direction to athletes is common practice. Likewise, in professional team sports, notational analysis codes the key contributions of each player during competition. Limited studies have however investigated relationships between physical capabilities and key performance indicators (KPIs) of rugby union match-play. Elite professional players, categorised as forwards (n = 15) or backs (n = 14), from an international rugby union squad (n = 29) undertook assessments of isometric mid-thigh pull (IMTP), bilateral and unilateral countermovement jumps (CMJ) and drop jumps (DJ; from 40 and 20 cm, respectively), and assessment of acceleration (10 m), a 5 m weighted sled drive, and a Yo-Yo intermittent recovery test level 1 (Yo-Yo IRTL1). Game statistics of the same players from 92 matches (~23 matches per player) during the 2014–15 season were analysed for effort and performance-based metrics. For forwards, Yo-Yo IRTL1 correlated significantly with; number of tackles made (r = 0.717), first three players at a ruck in both attack (r = 0.568) and defence (r = 0.581), number of effective rucks (r = 0.630), total possessions (r = 0.522), passes made (r = 0.651), percentage of carries over the gainline (r = 0.610), effective ruck success (r = 0.600), tackle success (r = 0.540), and the number of turnovers made (r = 0.518). Drop jump performance in forwards was associated with; the number of clean breaks (r = 0.558), dominant collisions (r = 0.589), and offloads (r = 0.594). For backs, the sled-drive test correlated with; number of carries (r = -0.751), first three players at an attacking ruck (r = -0.613), effective attacking rucks (r = -0.584), number of dominant collisions (r = -0.792) and offloads (r = -0.814). Likewise, for backs, IMTP peak force was related to; the number of possessions (r = 0.793), passes made (r = 0.792), effective attacking ruck percentage (r = 0.628), and the number of offloads (r = 0.621) whilst relative peak force correlated with; the percentage of carries over the gainline (r = 0.533), percent tackle success (r = 0.603) and effective attacking ruck percentage (r = 0.584). Regression analyses highlighted that only a small number of variables (i.e., carries, tackles, attacking and defensive first three at ruck) returned practically achievable changes (<20%) in physical qualities. In spite of this, and while leaving scope identification of further physical and/or performance predictors, greater strength, power and intermittent running performance were positively related to match-derived KPIs during competition. This may provide a basis for better integrating the strategies used by physical and technical performance-focused coaching staff to improve key performance indicators, and thus match performance, of rugby union players.

## Introduction

Rugby union currently employs two primary means of analysing match performances and quantifying the movement demands of professional players. Namely, notional analysis (to assess player actions) from camera-based systems, and global positioning system (GPS) devices worn by players (to assess movement demands). Numerous studies have reported the external workload of players across both union and league codes [1–4], and have demonstrated positional differences [1, 2, 5] and the impact of transient match-related fatigue [1, 2]. However, the array of physical outputs reported in such studies (e.g. distance covered, high speed running distance) have not always been associated with successful match outcomes [6] or even been highlighted as a distinguishing factor between playing standards [7]; thus more information on in-game actions is needed to provide context to the external workload.

Key performance indicators (KPIs) are therefore favoured by practitioners as they allow for quantification of the contribution of each player to specific areas of the game deemed integral to successful performance. Watson et al., [8] performed a retrospective and comprehensive analysis of the KPI literature in rugby union, and compiled a large data set of competitions, seasons and KPIs. Only 12 KPIs were significantly linked to performance across all competitions, and the KPIs that displayed medium or large effect sizes focused on try-scoring, territorial dominance and creation and successful execution of scoring opportunities when entering the opposition 22 m. However, these KPIs were deemed to be considered “common sense” in that they do not reveal anything novel to those involved in the game. Accordingly, further insight and practical impact of this type of KPI approach to research was called for with an emphasis on identifying specific variables that contribute to these KPIs.

A plethora of research highlights the importance of strength, power, aerobic and anaerobic fitness on indices of speed, change of direction, work rate, recovery and injury prevention in athletes [9–13]; all of which have been shown to underpin successful performance in a variety of sports [14–16]. In rugby union, researchers have reported significant correlations between relative squat strength and 10 m acceleration times (r = -0.55). Similarly, large to very large correlations have been reported between indices of countermovement jump (CMJ) and drop jump (DJ) performance (i.e., relative power output and jump height; JH) and sprint times over both the initial accelerative (10 m) and maximum velocity phases (flying 10 m from a rolling 20 m start) (r = -0.55 to -0.88; [9]). Whilst the relationship between performance on physical tests and movement capabilities are reasonably well established [9, 17–20], the link to physical qualities and game behaviours has yet to be fully explored. A deeper insight into KPIs will likely be of interest to coaches and applied practitioners whose game plans and playing styles are influenced by prerequisites of physical and technical performance.

Links between match analysis statistics and outcomes in rugby union have been reported [21–23]. Ortega et al., [22] reported that winning teams outperformed their opposition for mauls won, line breaks, possessions kicked, tackles completed, and turnovers won. Conversely, losing teams conceded significantly more set pieces (scrums and line-outs) in Six Nations international matches [22]. In seven’s rugby, moderate to large (-0.32–0.51) negative correlations between 10 and 40 m sprint times and the number of line breaks, defenders beaten and tackle effectiveness have been reported together with a moderate relationship between repeated sprint ability and maximal aerobic capacity and indices of in-game work rate such as the sum of tackles affected, effective attacking rucks and effective defensive rucks (~0.38; [20]). Notably, mixed-model analyses revealed that a decrease of two between-player standard deviations in 10 m sprint time (from 1.70 s to 1.54 s) would be associated with an increase of 74% more line breaks per match. However, whilst this information is beneficial for coaches, such magnitudes of decrease in sprint time may be unobtainable from a practical perspective.

Smart et al., [14] provide some of the limited data that has investigated the relationship between physical qualities and KPIs during match-play in rugby union. From 510 players and 296 matches, it was observed that sprint times over 10, 20 and 30 m had small to moderate negative correlations with the number of line breaks, metres advanced, tackle breaks and tries scored (r = -0.13 to -0.32). Activity rate (defined as any action performed by the player) was found to have moderate to small correlations with mean sprint time during a repeated sprint ability test in forwards (r = -0.38), and repeated sprint fatigue (r = -0.17) in backs. This type of investigation can provide objective data to concentrate on the development of certain physical qualities with the goal of maximising the transfer to on-field actions, performance, and ultimately success. However, these researchers did not include any measure of power in their testing battery and only included absolute values from isotonic strength tests which provide limited information and are heavily dependent on technique [24]. Therefore, the aim of the current study was to investigate the relationship between various physical performance tests (including a comprehensive strength and power testing battery) and key performance indicators during elite northern hemisphere rugby union match-play. In addition, regression analyses will attempt to predict what level of change in physical measures is required to improve match KPIs.

## Method

Senior elite (*n* = 29) professional players from an international rugby union squad (forwards; *n* = 15, age: 26 ± 3 years, height: 1.89 ± 0.06 m, weight: 115.8 ± 7.3 kg, backs; *n* = 14, age: 26 ± 3 years, height: 1.83 ± 0.05 m, weight: 94.5 ± 9.6 kg) participated in this study where physical testing was conducted at the end of the preseason period. Prior to providing written informed consent, players were informed of the rationale, potential applications, procedures and risks associated with the study. Ethics approval was granted by the Swansea University Ethics Committee and players were recruited on the basis that they had been selected for an elite international performance squad at the time of data collection (August 2014) and had engaged in a structured training program for at least 2 years beforehand. All players were in full-time training and thus considered healthy and injury-free as per the recommendations of the medical team. Players were provided with food throughout the camp in line with a nutritionist’s recommendations.

### Experimental approach to the problem

Before the start of the main experimental trials, players visited the laboratory to become familiar with the testing procedures of the study. Forty-eight hours after familiarisation, all players performed two testing sessions separated by 24 h of recovery. Participants reported to the laboratory on the first morning of testing having refrained from alcohol, caffeine, and strenuous exercise for 48 h. On the first testing day, following the collection of basic anthropometric measurements, players completed a standardised warm-up before a maximum isometric midthigh pull (IMTP) assessment, which was followed by bilateral and unilateral countermovement jumps (CMJ and SLCMJ, respectively), drop jumps from 40 cm (DJ40) and 20 cm (DJ20), single leg drop jumps from 20 cm (SLDJ20), 10 m sprints and 5 m sled drives. On the second day participants performed the Yo-Yo intermittent recovery test level 1 (Yo-Yo IRTL1) following the same standardized warm-up. Ad libitum consumption of 500 ml of water was permitted during each session and room temperature was maintained between 20 and 24°C. Verbal encouragement was given throughout testing by performance staff of the governing body and the research team to maximize performance. Ninety two matches incorporating the same group of participants were also analysed for performance metrics via notational analysis from the 2014–15 season which included English Premiership, European, Autumn International and Six Nations games.

### Isometric midthigh pull testing (IMTP)

The IMTP testing was carried out with participants standing on a portable force platform (type 92866AA, Kistler Instruments Ltd., Farnborough, United Kingdom), which was positioned in a custom rig centred under a bar. Participants were positioned so that they assumed a body position similar to that of athletes completing the second pull of a power clean with a flat trunk position and their shoulders in line with the bar. This position allowed athletes to maintain a knee angle of between 120–130° as previously used [25, 26]. The bar could be fixed at various heights above the force platform, to accommodate participants of different statures. Once the bar height was established, the participants stood on the force platform, with their hands strapped to the bar in accordance with previously established methods [25, 26], and were required to provide three acceptable trials. The portable force platform with built-in charge amplifier was used to measure the vertical component of the ground reaction force (GRF) of the subjects during performance of a maximal effort IMTP. A sample rate of 1,000 Hz which has been shown to be accurate and reliable when testing the IMTP [27] and a vertical force range of 20 kN were used for all trials. The force–time data were recorded on a portable computer using a 16-bit analogue to digital converter. A sample length of 16 s was used for all trials, 4 s of quiet standing phase with the IMTP being initiated after a 3 s countdown and lasting for approximately five seconds. A rest period of four minutes between trials was used. Trials were also rejected if excessive pretension or unloading was present or if the participants’ shoulders deviated excessively from the line of the bar or if subjects were unhappy with the attempt. The platform’s calibration was checked before and after each testing session. During each trial, subjects were instructed to pull the bar as hard and as fast as possible for a period of approximately five seconds. These commands were based on previous research indicating that the use of these instructions produces optimal results for peak force (PF) and peak rate of force development (PRFD) [28, 29].

A reliable start time or initiation of the IMTP was needed as a reference point for calculation of subsequent variables [30]. For full explanation of methods please see West et al., [19]. Briefly, the start time (Ts) of the IMTP was then defined as the instant that the first derivative exceeded the mean value plus five standard deviations (SD). The PF was determined from the vertical component of the GRF–time history and was defined as the peak produced during the IMTP minus the subject’s body mass. The F @ 100 and 200 ms was defined as the absolute value of the vertical component of the GRF minus the subject’s body mass 100 and 200 milliseconds after Ts. The coefficient of variation (CV) for Peak force under similar protocols has been shown to be <5% [30]

### Countermovement jump (CMJ)

For the measurement of lower body power output, subjects completed three CMJs on the portable force platform with two minutes rest between jumps. To isolate the lower limbs, subjects stood with arms akimbo [31, 32]. After an initial stationary phase of at least two seconds in the upright position for the determination of body mass, the subjects performed a CMJ, dipping to a self-selected depth and then accelerating upward in an attempt to gain maximum height. Subjects landed back on the force platform, and their arms were kept akimbo throughout the movement. The same protocol was used for the SLCMJ with subjects standing on one leg during the stationary phase and minimising the swing of the non-standing leg during the dipping and jumping phase.

The vertical component of the GRF as the subject performed the CMJ was used in conjunction with the subject’s body mass to determine the instantaneous velocity and displacement of the subject’s centre of gravity (CG) [31]. Instantaneous power was determined using the following the standard relationship:

$$\mathrm{Power}\;\left(W\right)=\mathrm{vertical}\;\mathrm{GRF}\;\left(N\right)\;x\;\mathrm{vertical}\;\mathrm{velocity}\;\mathrm{of}\;\mathrm{CG}\;\left({\mathrm{ms}}^{‑1}\right)$$

From this peak power output (PPO) and jump height (displacement of CG) were reported. For full methods of the countermovement jump analysis please refer to Owen et al., [33]. The coefficient of variation for PPO was 3.1%. Following a test for the normality of distribution, data were expressed as the mean ± SD.

### Drop jump (DJ)

For the measurement of reactive strength subjects completed three DJ trials each from heights of 20 cm (both double and single leg) and 40 cm (double leg only) onto a portable force platform, with two minutes rest between jumps. The lower limbs were again isolated by having arms akimbo. Subjects were instructed to stand on a plyometric box positioned directly next to the force plate. On the command ‘go’ subjects stepped off the box and landed on both feet (or single leg for SL DJ20) and immediately jumped as high as possible. Subjects were instructed to minimise ground contact time whilst jumping as high as possible (with no tucking of the knees) and to land back on the force plate landing straight legged (with only a slight bend at the knee). The reactive strength index (RSI) was calculated by dividing flight time (time interval between toe off and landing) by contact time (time interval between first contact and toe off). Reactive strength index has been shown to be reliable from both 20 and 40 cm (CV < 5%) [34]. Jump height was estimated using the equation: Jump Height (m) = 1/8 (Gravity (m·s^-1^) *(Flight time (s)^2^)).

### Sprint acceleration testing (10 m)

The time taken to cover a distance of 10 m from a stationary start was used as the measure of acceleration performance. After their usual warm-up routine supervised by a qualified strength and conditioning coach, athletes performed three trials of maximum effort over the 10 m distance on an indoor track with two minutes rest between trials. Athletes started in a two-point crouched position with their preferred foot forward on a mark 30 cm behind the start gate. Single beam timing gates (Brower Timing System, Salt Lake City, UT, USA) were set up at the start (0 m) and at 10 m. The subjects were instructed to run as fast as possible during the test and to make sure to run all the way through the finishing gate and the fastest time of the three trials was used for data analysis. Momentum was calculated by multiplying the subjects mass by the average velocity over the 10 m sprint. The coefficient of variation for acceleration times was 2.7%

### Sled drive test

The time taken to drive forwards a weighted (120 kg for forwards, 110 kg for backs) tackle sled (Varsity Tackle Maker, Rogers Athletic, MI, USA) over 5 m was used as a contact/collision performance test. Athletes performed three trials with two minutes rest between attempts, after their sprint acceleration testing. Athletes started in a two-point crouched position with their preferred foot forward on a mark 1 m behind the sled. Two acceptable trials with two minutes rest between attempts were undertaken. Single beam timing gates (Brower Timing System, Salt Lake City, UT, USA) were set up at the start (0 m) and at 5 m to time the movement of the sled. The subjects were instructed to hit and push the sled as fast as possible during the test and the fastest time of the three trials was used for data analysis. The CV for this test was found to be moderate (CV < 10%)

### Yo-Yo intermittent recovery test level 1 (Yo-Yo IRTL1)

The Yo-Yo IRTL1 was performed with the subjects completing two 20 m shuttle runs, interspersed with 10 seconds of active recovery. The speed of the shuttles increased as the test progressed and is controlled by audio signals dictating the time in which the shuttles need to be completed within [35]. The test starts at 10 km·h^-1^ and increased progressively with the players stopping of their own volition or until they twice failed to reach the line on the beep [36]. The distance ran was recorded for analysis. Previous research [37] has shown CV (4.6%) values for the Yo-Yo IRTL1 to be reliable.

### Game key performance indicators (KPIs)

Actions on, and around the ball, performed by each individual player from all club and international games were coded from video footage into a time and location stamped performance matrix which was collated by the governing body (RFU). The definitions of the game KPIs used for the analysis are included in Table 1. These variables were selected in conjunction with the international coaching staff based on their experience and how they perceived successful performance. An experienced (>5 years’ experience) analysis team coded all games and demonstrated a intra and inter tester reliability of (CV) <2.7%. Variables were split into performance and effort-based categories. Effort-based variables provide information of overall work rate (e.g. number of carries, tackles and rucks hit), whereas performance-based variables have been shown to have direct impact on match outcome (e.g. tries scored) or are contextualized by success/failure (e.g. tackle success). These variables were deemed to be important for successful match-play in rugby union [14, 15, 23]. Each variable was calculated for each individual player and was normalised to game time (Reported value = (observed value/minutes played) x 80). To reduce the high random variation in game statistics from players that come off the bench during the latter stages of the game, only players with game time ≥ 10 minutes were included in the analysis [14, 38]

**Table 1:** Operational definitions of match statistics used in analysis.

**KPI Variable—Performance**	**Definition**
Clean Break	Count of times a player in possession of the ball breaks the defensive line
Half Break	Count of times a player in possession of the ball breaks the defensive line but is tackled in the process of doing so
Tries Scored	Number of tries scored by a player
Tackle Success (%)	Percentage of tackles that were successful
Carries Over Gainline (%)	Percentage of carries made that were over the gainline (imaginary line that runs the width of the pitch through the middle of the last breakdown)
Dominant Collisions	Count of collisions (both in attack and defence) where the player makes ground after the collision
Effective Attacking Ruck (%)	Percentage of times players were effective at the breakdown as one of the first 3 support players to the ruck while their team is attacking
Turnovers	Count of times a player turns over the ball into an offensive situation from a defensive play
Offloads	Count of times the player made a successful pass in the process of being tackled
**KPI Variable—Effort**	**Definition**
Carries	Count of times a player carried the ball into contact
Effective Attacking Ruck	Count of times players were effective at the breakdown as one of the first 3 support players to the ruck while their team is attacking
Tackles	Count of tackles made by the player
Attacking First Three	Count of the times the player was in the first 3 support players to the ruck while their team is attacking
Defensive First Three	Count of the times the player was in the first 3 support players to the ruck while their team is defending
Total Possession	Count of times player is in possession of the ball
Passes	Count of times the player passes the ball

### Statistical analysis

Players were grouped according to position (forward or back). Means and SD were calculated for each physical test score and game statistic. Game statistics were separated in to effort-based (e.g. number of tackles made) and performance-based game statistics. Correlation coefficients (r) were calculated between physical tests and games statistics using SPSS software (version 22; SPSS Inc. Chicago, IL) with significance set at p ≤ 0.05. The magnitudes of the correlation coefficients were interpreted with Cohen’s scale: <0.1 = trivial, 0.1–0.3 = small, 0.3–0.5 = moderate and >0.5 = large [39]. Following the correlation, all variables with non-significant relationships or with correlations r<0.3 were disregarded from further analyses. Subsequently, to explain common variance between those relationships remaining and to understand the magnitude and direction of change for the predictor variables with respect to the dependent variable, simple regression analysis was performed for each of the relationships. Although, repeated simple regressions increase the chance of type one error, the ratio of sample size to predictor variables was insufficient to run any type of multiple regression. All results should be interpreted with this limitation in mind.

## Results

Statistically significant correlation coefficients between selected physical measures and match performance are shown in Tables 2–5, whereas Tables A and B in S1 File present the KPI values and physical test performance data. For forwards, performance in the Yo-Yo IRTL1 test and DJ test produced the greatest number of significant correlations. Specifically, intermittent running performance in the forwards correlated with the effort variables; number of tackles made, first three players at a ruck (both attack and defence), number of effective rucks, total possessions and passes made (r = 0.522–0.717). In addition, performance-based measures such as the percentage of carries over the gainline, effective ruck success, tackle success, and the number of turnovers made also correlated significantly with intermittent running performance (r = 0.518–0.610). Reactive strength was also shown to be an important quality in forwards, and drop jump test performances (i.e., SL DJ 20, DJ 20, and DJ 40 RSI) correlated with effort-based metrics; number of carries (SL DJ 20), attacking first three (DJ 20 & DJ 40), and defensive first three (DJ 20) (r = 0.545–0.695), in addition to the performance-based metrics; clean breaks, dominant collisions, and offloads (DJ 20) (r = 0.558–0.594). A similar pattern was observed in the forwards with a relationship reported for both attacking and defensive first three players, passes made and dominant collisions. In the forwards, however, no IMTP variable showed a significant relationship with any KPI.

**Table 2:** Forwards effort-based match activities and physical test correlation.

KPI	Carries	Tackles	Attacking First 3	Defensive First 3	Effective Attacking Rucks	Total Possession	Passes
Body Mass (kg)						0.616	0.606
**Speed/Collision**							
10m Momentum (kg·s^-1^)							0.524
5m Sled Drive (s)			-0.447	-0.525			-0.570
**Drop Jumps**							
40 cm RSI (au)			0.548				
40 cm JH (cm)		0.643	0.669	0.638	0.743		
20 cm RSI (au)			0.690	0.677	0.550		
20 cm JH (cm)		0.641	0.595	0.545	0.654		
SL (avg) 20 cm RSI (au)	0.695						
**Endurance**							
Yo-Yo IRTL1 (m)		0.717	0.568	0.581	0.630	0.522	0.651

**Table 3:** Forwards performance-based match activities and physical test correlations.

KPI	Clean Break	Half Break	Tries Scored	Carries Over Gainline (%)	Dominant Collisions	Effective Attacking Ruck (%)	Tackle Success	Turnovers	Offloads
**Speed/Collision**									
10m Sprint (s)		-0.718		-0.651					
5m Sled Drive (s)					0.705				
**CMJ**									
PPO (W)					0.600				
Relative PPO (W·kg^-1^)	0.549			0.654		0.621			
JH (cm)	0.526			0.699	0.575	0.649			
SL (avg) PPO (W)	0.534				0.568				
SJ JH (cm)				0.591		0.627			
**Drop Jumps**									
40 cm JH (cm)			0.625					0.890	
20 cm RSI (au)	0.558				0.589				0.594
20 cm JH (cm)		0.530		0.726		0.574		0.738	
**Endurance**									
Yo-Yo IRTL1 (m)				0.610		0.600	0.540	0.518	

**Table 4:** Backs effort-based match activities and physical test correlations.

KPI	Carries	Tackles	Attacking First 3	Defensive First 3	Effective Attacking Rucks	Total Possession	Passes
Body Mass (kg)						0.626	0.566
**Speed/Collision**							
10m Momentum (kg·s^-1^)						0.643	0.593
5m Sled Drive (s)	-0.751		-0.613		-0.584		
**CMJ**							
SL (avg) PPO (W)	0.556						
**Drop Jumps**							
40 cm RSI (au)				0.569			
20 cm RSI (au)				0.657			
SL (avg) 20 cm RSI (au)				0.571			
**IMTP**							
PF (N)						0.793	0.792

**Table 5:** Backs performance-based match activities and physical test correlations.

KPI	Clean Break	Half Break	Tries Scored	Carries Over Gainline (%)	Dominant Collisions	Effective Attacking Ruck (%)	Tackle Success	Turnovers	Offloads
Body Mass					0.915				
**Speed/Collision**									
10m Momentum (kg·s^-1^)					0.862				0.776
5m Sled Drive (s)					-0.792				-0.814
**CMJ**									
PPO (W)					0.749				0.691
SL (avg) PPO (W)					0.794				0.728
**Drop Jumps**									
40 cm RSI (au)	0.621		0.636						
20 cm RSI (au)			0.622			0.582			
SL (avg) 20 cm RSI (au)	0.619		0.610						
**IMTP**									
PF (N)						0.628			0.621
Relative PF (N·kg^-1^)				0.533		0.584	0.603		
F @ 250 ms (N)						0.589			

For backs, the greatest number of significant correlations were derived from the sled drive test and IMTP whilst intermittent running performance did not significantly correlate with any effort or performance-based KPIs. The sled hit test correlated with the effort-based metrics; number of carries, first three players at the attacking ruck, effective attacking rucks (r = 0.584–0.751) and performance based measures; number of dominant collisions and offloads (r = 0.776–0.862). Peak force in the IMTP test correlated with number of possessions and passes made (r = 0.792–0.793) as well as effective attacking ruck percentage and offloads (r = 0.621–0.628). Other IMTP variables, such as F @ 250 ms correlated with effective attacking ruck and relative peak force showing a relationship with the percentage of carries over gainline, tackle success and effective attacking ruck (r = 0.533–0.603).

Using standardised beta values from the simple regression to predict the effect of a change in a physical quality on match KPIs revealed a number of significant effects. Some of these required changes however, were deemed beyond the scope of improvement in that physical attribute. Relationships that were deemed practically achievable (i.e., <20%) for the forwards were as follows; to increase the number of tackles made by one, would require either a 12.6% increase in DJ 20 cm jump height, 14.9% increase in DJ 40 cm jump height or 11.7% improvement in Yo-Yo IRTL1. To increase the count (by one) of being one of the first three players at a defensive ruck, a 18.4% increase in DJ 20 cm jump height, 18.7% increase in DJ 40 cm jump height or a 17.5% improvement in Yo-Yo IRTL1 performance was required. Lastly a 7.2% increase in DJ 20 cm jump height, a 7.6% increase in DJ 40 cm jump height or a 7.8% improvement in Yo-Yo IRTL1 performance would increase the count (by one) of being one of the first three players at an attacking ruck. For the backs to increase the number of ball carries by one, a 14.0% increase in SL CMJ PPO or 6.8% improvement in sled hit time would be required.

## Discussion

The aim of the current study was to examine the relationship between commonly used physical performance tests and KPIs collected during competitive match-play to evaluate what (if any) physical capabilities underpin successful on field actions. The current study identified that the performance of the backs positional group in strength (IMTP) and reactive strength (DJ) tests had the most significant correlations with game KPIs. In the forwards, results from lower body power (CMJ), reactive strength (DJ) and intermittent running performance (Yo-Yo IRTL1) had the most meaningful correlations with match KPI data. Such findings may have important implications for applied practitioners when structuring position-specific training.

Measures of 10 m acceleration speed have previously been reported to show small to large relationships with KPIs in rugby union (e.g. line breaks and tries scored; [14, 15]). In the current investigation however, 10 m acceleration sprint time was only significantly correlated with half breaks and percentage of carries over the gainline in forwards. Although the correlation between line breaks and 10 m acceleration sprint times did not reach significance in our study, the correlation values of -0.21 and -0.35 for forwards and backs, represent previous findings reported for rugby union forwards (r = -0.26), backs (r = -0.25), and rugby seven’s players (r = -0.47) [14, 15]. In the current study, tries scored and 10 m sprint time also produced correlations of -0.25 and -0.41 for forwards and backs, respectively. These correlations are of a higher magnitude than previous studies [14, 15] between tries scored and 10 m sprint times. Momentum over 10 m showed large correlations with contact events in the backs (dominant collisions and offloads) which is in agreement with Ross et al., [15] who reported a moderate correlation (r = 0.31) with dominant tackles in their study in rugby seven’s players. In the current study, an ecologically valid test for contact ability was sought by the conditioning staff and they devised a 5 m timed weighted sled drive. To date, no data is available to compare with previous research, however, performance on this test negatively correlated with the number of dominant collisions for both backs and forwards and the number of carries for backs, potentially providing an objective measure of contact ability. Further investigation is therefore needed to corroborate these results; however, this simple field test could give useful insight in to the collision capabilities of international rugby union players.

Intermittent running performance in the forwards group was found to have a number of large positive correlations with both effort and performance-based match activities (Tables 2 and 3). Although previous research [10, 14, 15] has used different tests to assess running-based fitness qualities (i.e., repeated speed, multi-stage fitness, and maximal aerobic speed tests), similar patterns between running based fitness test outcomes and total distances covered, ruck effectiveness, work rate, activity rate and tackling have been reported. Interestingly no significant correlation was found between Yo-Yo performance and any match activity variable for the backs. This is in agreement with Smart et al., [14] showing only very small correlation between their fitness test measure (repeated sprint fatigue) and activity rate (r = -0.17), attributing their findings to the uniformly high scores in the backs. Our findings are in agreement, with higher intermittent running performance scores of the backs group (1683 ± 289 m) that demonstrated less variation between players compared to the forward group (1429 ± 363 m). However, match-play contains many other activities in addition to running (collisions, grappling, getting off the floor etc.) which result in a disassociation between purely running-based tests and match-play.

Relative peak power and JH (CMJ), single leg peak power (SL CMJ) and RSI (DJ 20) showed a large positive correlation with clean breaks in the forwards. For the backs, RSI in the DJ 40 and SL DJ 20 were the only two significant correlations identified with clean breaks. Producing a high RSI score requires the ability to express large amounts of force in very short time frames. Performance in DJ tests has previously been related to maximum speed in a mixed group (i.e., forwards and backs) of rugby union players, furthermore single leg drop jump performance has been related to change of direction ability [9, 40]. This may suggest the backs rely more on these qualities compared to power (or slower stretch shortening activities) in the forwards to break through the contact. All three DJ tests (40, 20 & SL 20 cm) correlated with the number of tries scored in the backs, again highlighting the potential link to high speed [9] and agility [40] as important factors in scoring tries in this group.

All IMTP strength measures in the forwards group did not produce any statistically significant correlations with any KPI. Conversely in the backs, peak force, relative peak force and force at 250 ms showed large positive correlation with activities such as carries over the gainline, percentage of effective attacking rucks, tackle success and offloads. Strength underpins the ability to express higher power outputs [41–43], rate of force development and agility performance [25, 44, 45]. Similar correlations have been reported for tackle score (r = 0.42) and dominant tackles (r = 0.47) with loaded CMJ in rugby seven’s players [15]

Our correlation analyses revealed numerous large and statistically significant relationships between selected physical test scores and match KPIs. Further, regression analysis highlighted that for most of the relationships, the magnitude and direction of change for the predictor variables with respect to the dependent variable was beyond that which can be achieved from training (i.e., >20%). For example, increasing SL DJ 20 RSI by 0.574 (au) would lead to an extra carry for the forwards group. This however would represent a ~50% increase in the groups score on this variable which is likely to be beyond the scope of improvement in an already elite population. Only a small number of variables returned practically achievable changes in physical qualities. For the forwards improvements in in Yo-Yo IRTL1 distance and JH in the DJ tests (7.2–18.7%) were linked with improvements (by one) in tackle count and chance of being one of the first three at attacking and defending rucks. For the backs improvement in 5 m sled drive time and SL CMJ PPO (6.8–14%) improved number of carries (by one). In elite level rugby union players, improvements of ~4% in power have been reported over the course of ~33 weeks (Pre to mid-season) [46]. Similar improvements in peak power output (4.4%) along with large improvements in strength (14.8%) have been reported during a preseason training period [47]. Research investigating longitudinal (3 years) changes in power as a result of a periodised training program in Australian rules football reported improvements of ~14% in peak power output [48]. Whilst resisted sled sprint training over an 8 week period improved acceleration performance by ~4%, taking the group average 10 m time from 1.77 to 1.70 s [49]. Further investigation is needed to ascertain whether improving physical performance will directly impact KPI performance during match-play.

Limitations of the current study must be acknowledged. The participants came from a single international performance squad (although these players represented 10 different Premiership clubs) and the number of participants (*n* = 29) meant that individual position analysis could not be conducted with appropriate statistical power; however, differentiation of forwards and backs was possible. Matches from four different competitions were used and not all KPIs have shown to be statistically significant across all competitions [8]. It is possible that superiority shown in certain KPIs augmented the performance in the physical tests. The regression analysis used supports the rationale that the physical qualities augment the performance (KPIs). In spite of the limitations this study both confirms and advances the conclusions of the so far limited literature in rugby union on the topic [14].

The findings from this study demonstrate that physical qualities correlate with numerous KPIs; a finding which may support a more holistic approach between performance and technical coaching staff to prioritise certain physical characteristics with the goal of improving specific KPIs. For the forwards group, jump performance in the CMJ (PPO and JH) and DJ (RSI and JH) and intermittent running performance were most related to match KPIs. For the backs group, measures of strength (IMTP) and DJ performance (RSI) were correlated to the greatest number of KPIs. Collectively the results support the premise that generally higher levels of strength, power, reactive strength and intermittent running performance are associated with higher match KPIs. Therefore, specific methods to improve these qualities may confer benefits to match performance via KPI influence. A novel aspect of this study was using regression analysis predictions to estimate what change in a physical characteristic would lead to a change in match KPIs.

## Supporting information

S1 File (Table A) Game KPIs for International, European, and Premiership level rugby union during 2014–2015 season. Actions are per 80 mins of play. (Table B) Physical performance test results.
(DOCX)
