# Supplementary material for: Relationships between physical qualities and key performance indicators during match-play in senior international rugby union players
Source: PLoS One. 2018 Sep 12;13(9):e0202811. doi: 10.1371/journal.pone.0202811 (PMC6135371; doi:10.1371/journal.pone.0202811)
Supplement: S1 File — (Table A) Game KPIs for International, European, and Premiership level rugby union during 2014–2015 season. Actions are per 80 mins of play. (Table B) Physical performance test results. (DOCX) [file pone.0202811.s001.docx]

**Table A. Game KPIs for International, European, and Premiership level rugby union during 2014-2015 season. Actions are per 80 mins of play**

| **KPI Performance Variable** | **Forwards** | **Backs** |
| --- | --- | --- |
| Clean Break | 0.1 ± 0.1 | 0.3 ± 0.2 |
| Half Break | 0.2 ± 0.1 | 0.30 ± 0.2 |
| Tries Scored | 0.1 ± 0.8 | 0.2 ± 0.2 |
| Tackle Success (%) | 92.6 ± 2.9 | 85.9 ± 5.1 |
| Carries Over Gainline (%) | 43.6 ± 8.5 | 61.0 ± 6.9 |
| Dominant Collisions | 0.7 ± 0.5 | 0.7 ± 0.6 |
| Turnovers | 0.4 ± 0.4 | 0.0 ± 0.1 |
| Offloads | 0.3 ± 0.2 | 0.6 ± 0.4 |
| **KPI Effort Variable** | **Forwards** | **Backs** |
| Carries | 6.0 ± 1.6 | 5.3 ± 2.1 |
| Tackles | 12.4 ± 2.9 | 7.5 ± 2.3 |
| Attacking First 3 | 24.7 ± 5.5 | 6.1 ± 3.4 |
| Defensive First 3 | 4.2 ± 2.3 | 1.3 ± 0.7 |
| Effective Attacking Ruck (%) | 93.1 ± 2.5 | 86.2 ± 10.7 |
| Total Possession | 9.6 ± 2.4 | 35.4 ± 26.0 |
| Passes | 2.8 ± 1.1 | 23.4 ± 24.7 |
| Offloads | 0.3 ± 0.2 | 0.6 ± 0.4 |

**Table B. Physical performance test results**

| **Physical Measure** | **Forwards** | **Backs** |
| --- | --- | --- |
| Body Mass (kg) | 115.8 ± 7.3 | 94.5 ± 9.6 |
| **Speed/Collision** |  |  |
| 10 m (s) | 1.8 ± 0.1 | 1.7 ± 0.1 |
| 10 m Momentum (au) | 638.4 ± 45.5 | 562.2 ± 52.2 |
| 5 m Sled Hit (s) | 1.6 ± 0.2 | 1.8 ± 0.2 |
| **CMJ** |  |  |
| Peak Power (W) | 5954.8 ± 713.5 | 5667.9 ± 1021.2 |
| Relative Peak Power (W/kg) | 51.4 ± 5.3 | 59.8 ± 7.6 |
| JH (cm) | 35.1 ± 5.4 | 41.8 ± 4.6 |
| Single Leg (avg) Peak Power (W) | 3462.6 ± 471.4 | 3393.3 ± 467.5 |
| SJ JH (cm)* | 34.7 ± 5.0 | 41.7 ± 4.6 |
| **Drop Jumps** |  |  |
| 40 cm RSI (au) | 2.1 ± 0.4 | 2.8 ± 0.6 |
| 40 cm JH (cm) | 26.8 ± 7.5 | 38.4 ± 4.9 |
| 20 cm RSI (au) | 2.1 ± 0.4 | 2.7 ± 0.6 |
| 20 cm JH (cm) | 26.3 ± 6.2 | 37.4 ± 6.3 |
| Single Leg (avg) 20 cm RSI (au) | 1.2 ± 0.2 | 1.5 ± 0.3 |
| **IMTP** |  |  |
| Peak Force (N) | 3569.3 ± 633.1 | 3361.2 ± 463.4 |
| Relative Peak Force (kg/kg BM) | 3.2 ± 0.6 | 3.6 ± 0.4 |
| Force at 100 ms (N) | 1301.3 ± 577.4 | 1477.6 ± 492.8 |
| Force at 250 ms (N) | 2282.0 ± 279.9 | 2244.9 ± 337.8 |
| **Aerobic Endurance** |  |  |
| Yo-Yo IRT L1 (m) | 1429.3 ± 363.3 | 1682.9 ± 289.1 |
